# Automated Segmentation of Retinal Pigment Epithelium Layers in High-Resolution OCT

**DOI:** 10.1016/j.xops.2026.101354

**Published:** 2026-08-04

**Authors:** Giulia Ritter, Ben Isselmann, Marlene Saßmannshausen, Anna S. Jauch, Katharina Wall, Philipp Herrmann, Frank G. Holz, Leon von der Emde, Thomas Ach

**Affiliations:** 1 Department of Ophthalmology, University Hospital Bonn, Bonn, Germany; 2 Division of Epidemiology and Clinical Applications, National Eye Institute, National Institutes of Health, Bethesda, Maryland

**Keywords:** Normative reference maps, Retinal imaging biomarkers, Thickness mapping

## Abstract

**Objective:**

To quantify retinal pigment epithelium (RPE) layer thicknesses and reflectivity in healthy participants across different age groups using improved axial, high-resolution (High-Res) OCT.

**Design:**

A cross-sectional observational study.

**Subjects:**

A total of 110 eyes from 110 healthy participants were included. Participants were categorized into 3 age groups: group 1 (<40 years), group 2 (40–65 years), and group 3 (>65 years).

**Methods:**

High-resolution OCT imaging was performed, followed by the segmentation of 3 RPE layers. The inner layer corresponded to RPE layer 1 (High-Res OCT nomenclature) and outer segment interdigitation zone 2, the middle RPE layer corresponded to RPE layers 2 and 3, and the outer RPE layer corresponded to RPE layers 4 and 5 and Bruch membrane. A convolutional neural network (CNN) was trained for automated RPE layer recognition. Volumetric thickness maps and reflectivity values were generated to assess age-related differences across the macula and analyzed using the Kruskal–Wallis and Mann–Whitney *U* tests.

**Main Outcome Measures:**

The primary outcome measures were RPE thicknesses and vitreous-normalized reflectivity of the 3 RPE layers across the 3 age groups.

**Results:**

The CNN achieved high segmentation accuracy with a mean Dice of 0.85. Hyporeflective RPE layer 1 was absent at the fovea but increased in thickness with eccentricity. Significant age-related increases were observed in the reflectivity throughout the middle hyperreflective RPE layer, at the fovea (H = 13.8; *P* = 0.001), the inner ETDRS ring (H = 19.8; *P* < 0.001), and the outer ETDRS ring (H = 16.2; *P* < 0.001). For the outer hyperreflective RPE layer, an increase in thickness with age was noted in the outer (H = 8.63; *P* = 0.013), along with an increased reflectivity at the fovea (H = 14.8; *P* < 0.001) and the inner ETDRS ring (H = 14.35; *P* < 0.001).

**Conclusions:**

OCT imaging with improved resolution enables detailed segmentation of RPE layers, revealing moderate but significant age-related changes in reflectivity and thickness. These findings provide normative data essential for identifying early retinal alterations and developing structural biomarkers for age-related retinal diseases.

**Financial Disclosure(s):**

Proprietary or commercial disclosure may be found in the Footnotes and Disclosures at the end of this article.

The retinal pigment epithelium (RPE) plays a crucial role in maintaining retinal homeostasis, supporting photoreceptor function, and regulating the visual cycle.^1^^,^^2^ In conventional spectral-domain OCT imaging, the RPE appears as a hyperreflective band because of its light-scattering properties, which are influenced by organelles such as mitochondria, melanosomes, and lysosomes (lipofuscin and melanolipofuscin) within RPE cells.^3^^,^^4^

Advances in high-resolution (High-Res) imaging technologies, however, have enhanced our ability to study the RPE in greater detail.^5^, ^6^, ^7^, ^8^ Imaging techniques such as High-Res OCT and adaptive optics OCT display a complex, multilayered RPE structure rather than a uniform hyperreflective band.^9^, ^10^, ^11^ Notably, a study by Goerdt et al^11^ has demonstrated the ability of High-Res OCT to resolve up to 5 distinct RPE layers, each corresponding to different cellular components and subcellular structures. These 5 layers are thought to correspond to (1) the apical processes of the RPE, which interact with photoreceptor outer segments; (2) the central region rich in melanolipofuscin and lysosomal organelles; (3) an intermediate region associated with relatively sparse organelle density; (4) the basal third of the cell body packed with mitochondria; and (5) the basal infolding labyrinth, which interfaces with Bruch membrane (BM) and also could reflect basal laminar deposit (BLamD), if present.^12^, ^13^, ^14^, ^15^ However, the same study also highlights the limitations of accurate layer recognition, particularly in aging and disease states.^11^ The ability to distinguish all 5 RPE layers (using magnification and reflectivity plots on an OCT A-scan level) was limited to a subset of participants (in healthy participants, between 43.1% and 82.0%, dependent on age; in age-related macular degeneration (AMD), 46.9%, from 17 participants with early AMD and 15 participants with intermediate AMD).^11^ To expand on this, the visibility of the hyporeflective RPE layer 5 (between hyperreflective RPE layer 4 and BM) could drop below 20% on some grading positions in aged participants, showing that a distinction between the 2 is not practical with current, although enhanced, lateral resolution.

A prerequisite for improved diagnostic accuracy to facilitate automated layer recognition is the application in large data sets and longitudinal monitoring of RPE integrity. In these settings, we propose focusing on easily delineable hyperreflective bands within the RPE.^16^^,^^17^ Upon visual inspection without magnification, the following 3 hyperreflective bands can be easily delineated using High-Res OCT imaging: inner (outer segment interdigitation zone 2 and RPE layer 1 outside the foveal bouquet), middle (RPE layer 2), and outer layer (BM and RPE layer 4) (Fig 1).^18^

**Figure 1:**
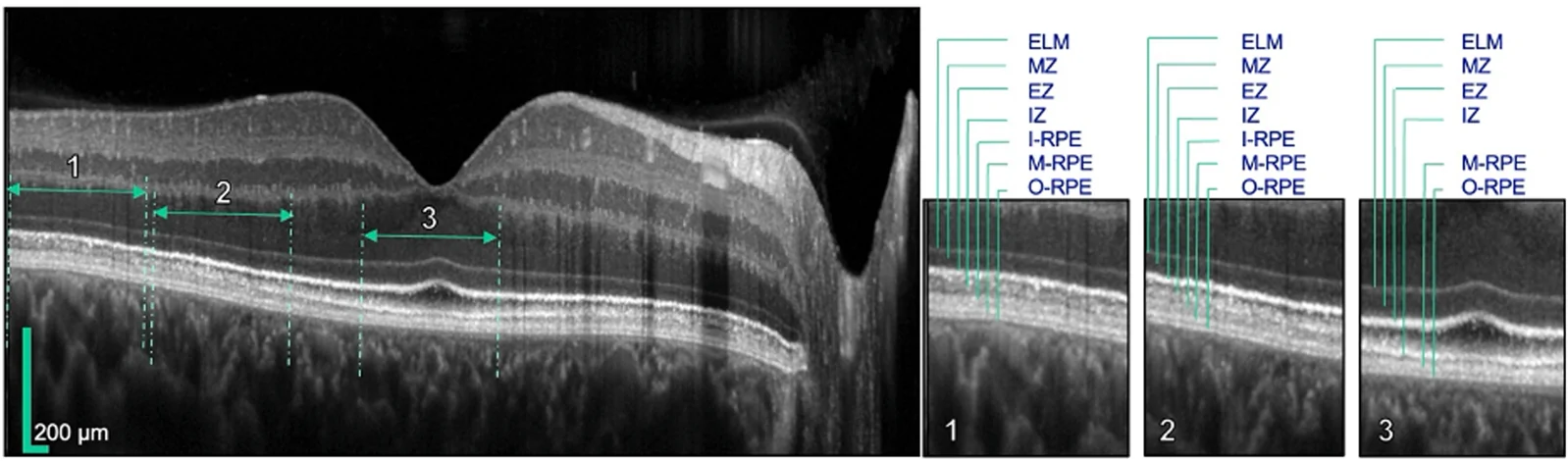
High-resolution OCT image through the fovea. Retinal layers of a healthy retina in a central B-scan (algorithmic depiction) of a high-resolution OCT image. Close-up views of the outer retina with the 3-layered RPE complex, among others, at 3 retinal locations (1 = centered ∼5500 μm from the foveal center; 2 = centered ∼4000 μm from the foveal center; and 3 = centered on the foveal center. ELM = external limiting membrane; EZ = ellipsoid zone; I = inner; IZ = interdigitation zone; M = middle; MZ = myoid zone; O = outer; RPE = retinal pigment epithelium.

This study aims to quantify RPE layer thicknesses and reflectivity in healthy participants of varying ages. Establishing normative data on a simplified RPE nomenclature for automated analysis could enable the detection of subtle RPE changes associated with aging and early-stage retinal diseases. Moreover, these findings could contribute to the development of structural biomarkers for retinal functional assessments, facilitating early diagnosis and targeted interventions for degenerative retinal conditions.

## Methods

### Participants

Healthy participants were recruited from the Department of Ophthalmology, University of Bonn, Germany, between December 2023 and December 2024. Inclusion criteria were at least 18 years of age and no prior intraocular surgery of the eye, with the exception of cataract surgery (at least 3 months before examination). Exclusion criteria included refractive errors ≥3.00 diopters (D) (spherical) and >1.50 D (cylindrical) assessed by autorefraction (ARK-560A; Nidek), as well as a history of any retinal disease, glaucoma, or relevant anterior segment diseases with media opacities. OCT images were reviewed by a retinal specialist (L.v.d.E.; senior grader, GRADE reading center) and excluded in the presence of droplets or other signs of subclinical retinal alterations. If both eyes met the inclusion criteria, 1 eye was randomly picked for analysis. All participants underwent routine ophthalmic examinations, including best-corrected visual acuity, slit lamp, and funduscopic examination. The institutional review board of the University of Bonn approved the study (ethics approval ID: 305/21), and all participants gave written informed consent after explaining the nature of the study. All study procedures adhered to the tenets of the Declaration of Helsinki.

### Imaging Protocol and Analysis

Standardized retinal imaging was performed combining confocal laser ophthalmoscopy and High-Res spectral-domain OCT (High-Res OCT) imaging (30° × 25°, ART 25, fast and High-Res mode, 121 B-scans, axial resolution: 2.9 μm, wavelength 853 μm, power: 2.2 mW; Heidelberg Engineering).

Volumetric High-Res OCT data were manually segmented in a subset of patients (*n* = 25 participants; 705 B-scans [5 patients, 121 B-scans; 20 patients, 5 B-scans each]; 2 graders [G.R. and L.v.d.E.] as implemented in the manufacturer’s software [Spectralis Viewing Module 6.3.2.0; Heidelberg Engineering] for algorithm training). Because different RPE layers are not yet included in the manufacturer’s software package, the photoreceptor layers were misclassified (different layers reused after download and renamed within Python; Python 3.10, Python Software Foundation) to create annotations (Figs 1, S1, available at www.ophthalmologyscience.org). Manual segmentation was performed on logarithmically scaled OCT B-scan images, enhancing contrast to facilitate clearer visualization of the retinal layers for the graders.

In total, 3 RPE bands were annotated (i.e., 3 hyperreflective layers, when visible). The inner RPE layer ranged from the outer segment interdigitation zone to RPE layer 2, the middle hyperreflective RPE layer ranged from RPE layers 2 to 4, and the outer hyperreflective RPE layer ranged from RPE layer 4 to BM, based on the recently proposed High-Res OCT nomenclature (Table 1).^11^

**Table 1:** OCT-Based Layer/Band Nomenclature

Nomenclature in this Study	High-Res OCT Nomenclature^11^	Histological Correlate^11^
Inner hyperreflective RPE layer	Outer segment interdigitation zone 2 Hyporeflective RPE layer 1	Apical processes of RPE or rod outer segment tips
Middle hyperreflective RPE layer	Hyperreflective RPE layer 2	Densely packed lysosome-related organelles
	Hyporeflective RPE layer 3	Reduced organelle density
Outer hyperreflective RPE layer	Hyperreflective RPE layer 4	Dense aggregation of mitochondria
	Hyporeflective RPE layer 5	Basal infoldings or basal lamina deposits
	Bruch membrane	Bruch membrane

High-Res = high-resolution; RPE = retinal pigment epithelium.

Thereafter, the segmentations were used for the training of a convolutional neural network (CNN; DeepLabv3 model; Google Research/Google Brain; see details below) and were thereafter applied to the entire data set (>13 000 B-scans from 112 participants). Volumetric thickness maps for each retinal layer were generated for illustrative purposes by averaging thickness measurements across all participants. These maps were created using Fiji (ImageJ; version 2.4.0) and computed on a voxel-by-voxel basis by calculating the distance between corresponding upper and lower segmentation boundaries at each x/y position across the scan volume. Furthermore, the RPE layer reflectivity for each of the 3 hyperreflective layers within the ETDRS grid was extracted for analysis by the CNN from “.VOL” files. The CNN analyzed B-scans with their unprocessed signal intensities of 0 to 255 (no logarithmic transformation) and normalized these to values between 0 and 1. The median vitreous reflectivity, measured ≥10 pixels above the inner limiting membrane, was used as a stable reference layer for normalization.^19^^,^^20^ Normalized, patient-individual vitreous reflectivity values were extracted from unprocessed “.XML” files using Fiji (ImageJ; version 2.4.0) and then subtracted from the previously assessed RPE reflectivity (RPE – vitreous). A ratio-based adjustment would artificially exaggerate small changes as vitreous reflectivity approaches 0 (reflectivity/0 = infinity). However, the raw, unprocessed image format with linear pixel intensities allowed normalization by subtraction.

### Statistical Analysis

Statistical analyses were conducted using R (version 4.3.1) for general statistical computations and Python (versions 3.10 and 3.12.13; Python Software Foundation) with the frameworks PyTorch (version 2.0.1) and MONAI (version 1.2.0) for neural network training. Statistical analyses were performed with SciPy for Kruskal–Wallis and Mann–Whitney *U* tests, and with statsmodels for multiple comparison correction. The DeepLabV3 (Google Research/Google Brain) model was used. The process used the Adam optimizer with a learning rate of 10^–5^. Model performance was evaluated using fivefold cross-validation, and metrics (dice similarity coefficient) and Intersection over Union [IoU]) were calculated on each fold’s test set and then averaged across all folds. Retinal pigment epithelium layer thicknesses and the vitreous-normalized RPE reflectivity were reported as means and standard deviations (SDs) across the ETDRS grid. Age-related differences were compared with the Kruskal–Wallis test between the 3 groups (*P* and H test statistic values are reported) and followed up with the Mann–Whitney *U* test (*P* and *U test* statistic values reported) after confirming a nonnormal distribution. A *P* value below 0.05 was considered statistically significant, and subgroup *P* values were adjusted using the Bonferroni correction.

## Results

### Patient Cohort

A total of 110 eyes of 110 healthy participants were included in the study (Table 2). The age range spanned from 21 to 87 years, and, for the analysis, the cohort was divided into the following 3 age groups: group 1 (<40 years; *n* = 25; mean ± SD = 28.8 ± 5.0), group 2 (40–65 years; n = 35; mean ± SD = 55.7 ± 7.3), and group 3 (>65 years; n = 50; mean ± SD = 74.0 ± 6.13). The subgroup of manual retinal layer annotations consisted of 25 participants (*n* = 16 female; *n* = 9 male; age, 56.9 ± 21.3 years; range, 21–87 years).

**Table 2:** Characteristics of Study Population

Characteristic	Number
Participants, *n*	110
Eyes*,n*	110
Age (yrs), mean ± SD	58.6 ± 19.7
Group 1 [21–40, *n* = 25]	28.8 ± 5.0
Group 2 [40–65, *n* = 35]	55.7 ± 7.3
Group 3 [65–87, *n* = 50]	74.0 ± 6.1
Sex	
Male, *n* (%)	57 (51.8)
Female, *n* (%)	53 (48.2)

Data presented are means and SD, where applicable. SD = standard deviation.

### Accuracy of Retinal Layer Quantification

The accuracy of layer quantification for the 3 defined RPE layers showed consistently high performance, with a mean Dice score of 0.84 ± 0.03. Similarly, the mean IoU was 0.76 ± 0.03. Retinal pigment epithelium layer segmentation accuracy did not differ significantly (*P* > 0.3) among the 3 layers and participants.

### Inner Hyperreflective RPE Layer

The inner hyperreflective RPE layer could not be delineated at the fovea. It first appeared ∼600 μm from the foveal center and steadily increased in thickness with increasing horizontal and vertical eccentricity (Fig 2, Table 3). No statistically significant differences were found among the age groups for layer thickness (*P* = 0.09); however, reflectivity showed a statistically significant difference in the inner ETDRS ring (H = 8.01; *P* = 0.02), with subgroup analysis revealing a significant increase between groups 1 and 3 (*U* = 382.5; *P* = 0.048).

**Figure 2:**
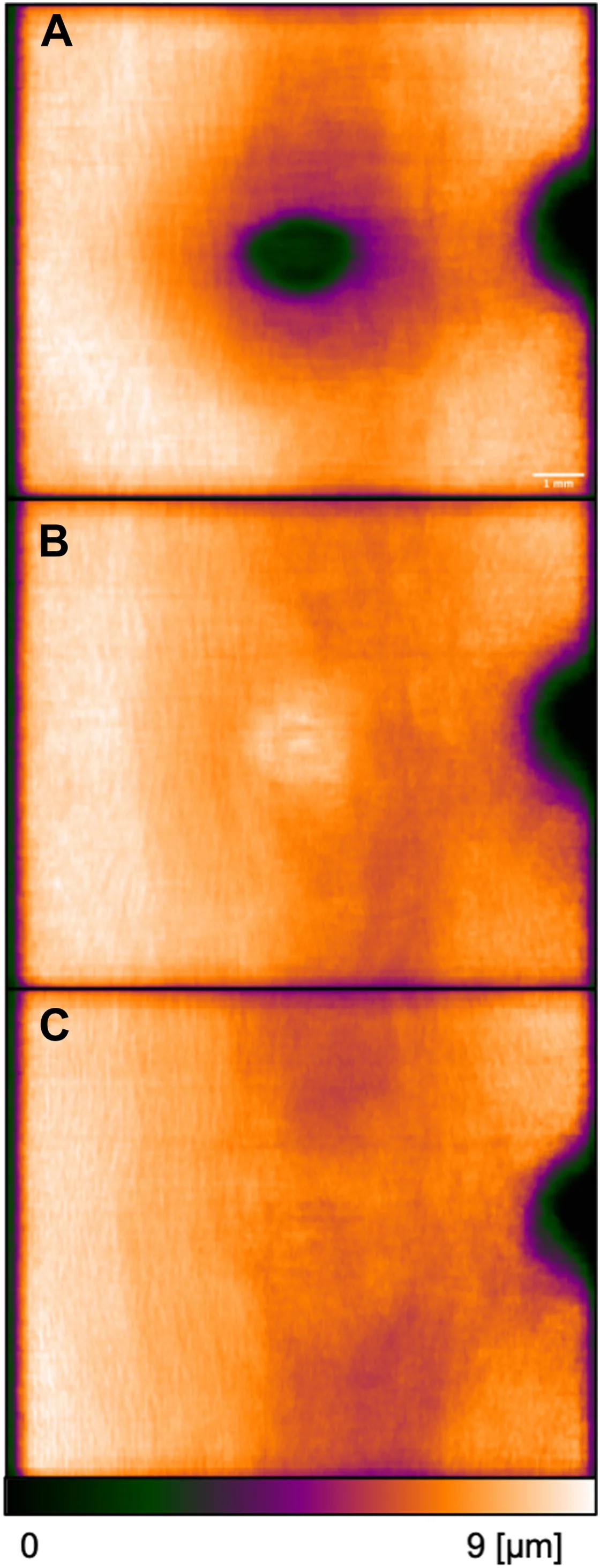
Retinal thickness maps of the retinal pigment epithelium (RPE) layers. Color-coded en face thickness maps of 3-layered RPE (inner hyperreflective RPE layer [**A**], middle hyperreflective RPE layer [**B**], and outer hyperreflective RPE layer [**C**]) in the macula (average of all participants: 30° × 25° and aligned with the optic nerve head and fovea). Left eyes were flipped horizontally for illustrative purposes. RPE layer 1 could not be identified within the foveal bouquet. Conversely, the middle RPE layer was slightly thicker within the fovea.

**Table 3:** Inner Hyperreflective RPE Layer Thickness and Ratio in ETDRS Subfields

ETDRS Subfield		Center	Inner Ring	Outer Ring
Group 1	Thickness	3.2 ± 2.5 μm	7.2 ± 1.3 μm	9.8 ± 1.2 μm
	Reflectivity	0.14 ± 0.09 a.u.	0.11 ± 0,06 a.u.	0.09 ± 0.05 a.u.
Group 2	Thickness	3.7 ± 2.8 μm	7.2 ± 1.6 μm	9.2 ± 1.5 μm
	Reflectivity	0.10 ± 0.07 a. u.	0.12 ± 0.06 a.u.	0.08 ± 0.04
Group 3	Thickness	4.5 ± 2.7 μm	7.5 ± 1.6 μm	9.3 ± 1.1 μm
	Reflectivity	0.13 ± 0.08 a.u.	0.15 ± 0.09 a.u.	0.08 ± 0.05 a.u.

This table summarizes the thicknesses and normalized reflectivity (reflectivity values subtracted by personal median vitreous reflectivity) of the inner hyperreflective retinal pigment epithelium layer across 3 ETDRS grid subfields: center, inner, and outer. Data are presented for 3 age groups: group 1 (<40 years), group 2 (40–65 years), and group 3 (>65 years). The values are expressed as means ± standard deviations in micrometers (μm) for thickness and arbitrary units (a.u.) for reflectivity. RPE = retinal pigment epithelium.

### Middle Hyperreflective RPE Layer

Across the ETDRS grid, the thickness of the middle hyperreflective RPE layer showed no age-related change. By contrast, the reflectivity showed significant age-related changes in all areas of the ETDRS grid. An increase in reflectivity was observed between groups 1 and 3, both in the central subfield (H = 13.77; *U* = 219.5; *P* = 0.0008) and in the outer ETDRS ring (H = 16.24; *U* = 301.5; *P* = 0.0008). The outer ETDRS ring also showed significant change between groups 1 and 2 (H = 16.24; *U* = 236; *P* = 0.008). The inner ETDRS ring of the middle hyperreflective layer showcased a significant increase in reflectivity (H = 19.85; *P* = 0.00005), with subgroup analysis showing change between all groups, indicating a stepwise significant increase in reflectivity from group 1 to group 2 to group 3 (Table 4; all Bonferroni-adjusted *P* < 0.025).

**Table 4:** Middle and Outer Hyperreflective RPE Layer Thicknesses and Ratios in the ETDRS Subfields

ETDRS Subfield			Center	Inner Ring	Outer Ring
Middle hyperreflective RPE layer	Group 1	Thickness	8.9 ± 3.7 μm	8.2 ± 1.7 μm	7.9 ± 0.7 μm
		Reflectivity	0.25 ± 0.12 a.u.	0.20 ± 0.10 a.u.	0.22 ± 0.08 a.u.
	Group 2	Thickness	9.6 ± 3.4 μm	8.3 ± 2.0 μm	8.2 ± 1.1 μm
		Reflectivity	0.34 ± 0.16 a.u.	0.28 ± 0.12 a.u.	0.28 ± 0.09 a.u.
	Group 3	Thickness	8.9 ± 2.1 μm	8.4 ± 1.3 μm	8.2 ± 1.2 μm
		Reflectivity	0.38 ± 0.16 a.u.	0.34 ± 0.14 a.u.	0.30 ± 0.12 a.u.
Outer hyperreflective RPE layer	Group 1	Thickness	7.8 ± 3.2 μm	8.0 ± 1.9 μm	7.7 ± 0.8 μm
		Reflectivity	0.13 ± 0.09 a.u.	0.13 ± 0.07 a.u.	0.19 ± 0.06 a.u.
	Group 2	Thickness	7.9 ± 2.2 μm	7.5 ± 1.8 μm	8.1 ± 0.7 μm
		Reflectivity	0.17 ± 0.10 a.u.	0.20 ± 0.12 a.u.	0.20 ± 0.7 a.u.
	Group 3	Thickness	7.8 ± 2.4 μm	8.2 ± 1.5 μm	8.1 ± 0.8 μm
		Reflectivity	0.23 ± 0.11 a.u.	0.21 ± 0.09 a.u.	0.21 ± 0.09

This table details the thicknesses and normalized reflectivity (reflectivity values subtracted by personal median vitreous reflectivity) of the middle and outer hyperreflective RPE layers across the ETDRS grid: center, inner ring, and outer ring. Data are presented for 3 age groups: group 1 (<40 years), group 2 (40–65 years), and group 3 (>65 years). Values are plotted as means ± standard deviations in μm for thickness and arbitrary units (a.u.) for reflectivity. RPE = retinal pigment epithelium.

### Outer Hyperreflective RPE Layer

The outer hyperreflective RPE layers showed age-related differences in both reflectivity and thickness (Table 4). Kruskal–Wallis tests revealed significant differences in the thickness of the outer hyperreflective RPE layer at the outer ETDRS ring (H = 8.63; *P* = 0.013) with post hoc Mann–Whitney *U* tests revealing that the thickness of the layer in the outer ETDRS ring was significantly lower in group 1, with a notable incline in group 3 (*U* = 335.0; *P* < 0.01). Reflectivity showed a significant change in the central subfield (H = 14.81; *P* = 0.0006), which was statistically significant between groups 1 and 3 (*U* = 219.5; *P* = 0.0008). Subgroup analysis of the inner ring showed a significant increase between groups 1 and 2 (*U* = 195; *P* = 0.03) and groups 1 and 3 (*U* = 239.5; *P* = 0.0006; Table 4).

## Discussion

The results of this study highlight the capacity of High-Res OCT imaging to resolve distinct structural layers within individual retinal layers. For the RPE, our findings confirm the presence of a hyperreflective RPE layer outside the foveal bouquet, as well as 2 hyperreflective layers, which were consistently identifiable across the macular High-Res OCT scans in healthy participants. We demonstrate slight but significant age-related thickness changes in both the middle and outer hyperreflective layers (but not the inner hyperreflective layer), highlighting structural integrity during healthy aging. The ability to segment these layers with high accuracy (Dice score of 0.84) further supports the feasibility of automated RPE analysis for future clinical applications. These findings underscore the importance of assessing individual RPE layers as potential structural biomarkers for age-related retinal changes and early disease detection.

Our measurements are in good agreement with histological findings, supporting the anatomical validity of High-Res OCT segmentation. The apical processes (inner RPE layer/RPE layer 1; High-Res OCT nomenclature) exhibited a thickness of ∼8 to 9 μm in our data set, slightly exceeding earlier reports of ∼7 μm.^2^^,^^10^ This layer was absent at the foveal center up to ∼600 μm eccentricity, accounting for markedly reduced values within the central ETDRS field.^10^ Previous studies have described considerable variation in the length of individual microvilli, with some processes extending beyond this range.^21^ Pollreisz et al^13^ reported vertical extents of the apical processes up to ∼15 μm, noting that the delicate structure of the apical processes and interdigitation with photoreceptor outer segments often obscure full visualization in postmortem specimens.

Regarding the RPE cell body, our layer-based segmentation yielded mean thicknesses of 8.1 to 9.1 μm for the middle RPE layer across the central, inner, and outer ETDRS rings, respectively. For the outer RPE layer (RPE 4 and 5, including BM; High-Res OCT nomenclature), we measured thicknesses of 7.8 to 8.0 μm (central, inner, and outer ETDRS rings), resulting in a total RPE cell body thickness of 15.9 to 17.0 μm (thickness of middle RPE layer + thickness of outer RPE layer).^11^ These results align well with electron microscopy studies by Pollreisz et al,^13^ who reported a mean apical-to-basal cell body distance of 15.8 ± 1.0 μm in 8 RPE cells examined.^13^ The combined thickness of the hyperreflective RPE layers of the cell body closely matches histological reference values (BM contributing ∼2–4 μm), thereby supporting the anatomical accuracy and validity of our automated measurement approach.^22^^,^^23^ The minor discrepancies of 1 to 2 μm lie comfortably within the reported SD and should be viewed in light of the fact that the above-cited histology was derived from only a few perifoveal donor RPE cells, a limitation inherent to the highly labor-intensive tissue processing required.

There is ongoing debate regarding the histological origin of the inner RPE layer. One hypothesis suggested that the hyporeflective RPE layer 1/outer segment interdigitation zone 2 represents a split in the interdigitation zone, in which cone outer segment tips and rod outer segment tips form 2 distinct bands because of the greater length of rod outer segments.^18^^,^^24^, ^25^, ^26^ This interpretation was supported by the absence of rod outer segment tips in the rod-free fovea.^27^ However, recent work by Goerdt et al^11^ challenges this notion, arguing that cone and rod outer segment lengths may be similar in humans—a discrepancy that has only been demonstrated in mice and frogs so far.^11^^,^^28^ Additionally, ultra-High-Res OCT imaging has revealed that the so-called rod outer segment tips layer extends almost to the foveal center, further questioning the traditional view.^9^^,^^29^^,^^30^ Although the present study does not definitively resolve this issue, the thickness maps (Fig 2) of the inner RPE layer here seem to mimic maps of rod density from the literature.^27^ Moreover, unpublished observations by our group using adaptive optics indicate that solitary rods persist up to half a degree eccentricity from the foveal center (personal communication with Julius Ameln, AOLab, University of Bonn, 2024). To conclusively determine the nature of this layer, further studies combining ultra-High-Res imaging with histological correlation are required.

The middle RPE layer demonstrated rather constant RPE layer thickness during normal aging. Solely, the reflectivity showed a significant increase in aging patients. Although the OCT signal is often attributed to densely packed mitochondria, the cytoplasmic load of RPE granules (melanosomes, lipofuscin, and melanolipofuscin) also makes a substantial contribution to its reflectivity. This is evidenced by the fact that the middle RPE layer, which anatomically is located more apically than the mitochondria-rich basal third of the cell, remains highly reflective on High-Res OCT despite its relative paucity of mitochondria. Accordingly, variations in granule density and composition may contribute to layer-specific reflectivity profiles and thickness measurements in aging and disease, although this cannot be determined directly from OCT alone. Our previous histological studies showed stable autofluorescent granule counts within the RPE cell body.^12^^,^^31^ Furthermore, the increased reflectivity observed in this study may correspond to subtle changes in granule composition or optical density, possibly related to the accumulation of both melanolipofuscin and lipofuscin, which is shown to slightly increase with age, whereas melanosomes decrease.^31^^,^^32^ It is noteworthy that the density of intracellular melanolipofuscin granules reaches its maximum value in the center of the fovea, which corresponds to the location where we measured the highest reflectivity density.^30^^,^^31^

The outer RPE layer exhibited a slight increase in both thickness and reflectivity, likely because of multiple contributing factors. Our definition of the outer RPE layer encompasses several histological components, including BM, BLamD (if present), basal infoldings, and the basal RPE cell body (particularly composed of mitochondria). Bruch's membrane, for instance, has been shown to thicken threefold with aging, with the most pronounced changes occurring in the central macula compared with peripheral regions.^8^^,^^22^^,^^33^^,^^34^ Age-related calcification, characterized by fine deposits of calcium phosphate on individual elastin fibrils, represents one possible explanation for the observed increase in reflectivity, although this interpretation remains speculative in the absence of direct histological correlation.^35^^,^^36^ Additionally, age-related accumulation of BLamD could contribute to increased thickness, although it may not fully explain the observed rise in reflectivity. Basal laminar deposit, consisting of basement membrane proteins and long-spacing collagen, is located between the RPE basement membrane and its plasma membrane.^33^^,^^37^ These deposits are visible on OCT as a hyporeflective layer in a split RPE-BL-BrM complex and are found in both normal aging eyes and those affected by AMD.^38^ In AMD, BLamD can increase the thickness of the RPE-BLamD complex (depending on the study) up to 30 μm (5.5 μm on average).^39^ Furthermore, changes within the RPE cell body and mitochondrial composition may also be associated with these findings, but the present imaging data do not allow direct attribution of the observed signal changes to specific subcellular structures. Mitochondrial numbers within the RPE, however, decrease with age.^40^ Aging mitochondria are more susceptible to morphological alterations resulting from oxidative damage, potentially affecting reflectivity.^41^ Conversely, it is worth noting that mitochondrial changes within photoreceptors, particularly in the ellipsoid zone, are typically associated with decreased reflectivity in OCT imaging in the context of aging and AMD.^42^ Further studies combining High-Res OCT with histological validation are needed to fully elucidate the underlying mechanisms of these age-related changes.

This study provides a comprehensive analysis of RPE layer segmentation using High-Res OCT imaging, contributing significantly to our understanding of retinal structural changes associated with aging. One of its key strengths lies in the high segmentation accuracy achieved, with a mean Dice score of 0.923 and a mean IoU of 0.860, highlighting the reliability and reproducibility of the proposed CNN model. Additionally, the study’s ability to consistently identify distinct hyperreflective bands across a broad age range enhances its generalizability and clinical relevance. The use of High-Res OCT to subdivide the RPE layer shows potential for novel structural biomarkers for early detection of retinal diseases. However, certain limitations should be acknowledged. The manual annotation of retinal layers, although meticulous, was performed on a small subset of participants only, potentially introducing selection bias and limiting the training data for the neural network. Furthermore, the cross-sectional design of the study precludes conclusions about longitudinal changes in RPE layer thickness and reflectivity. The study faced challenges in resolving all 5 RPE layers from the recently proposed High-Res OCT nomenclature, even in the absence of retinal diseases.^11^ It is therefore plausible that even the simplified 3-layer segmentation cannot be achieved with diagnostic accuracy in a clinical setting where disease-induced structural changes are more pronounced. Furthermore, this study identifies 2 hyperreflective RPE layers that could theoretically correspond to 3 hyperreflective structures of the High-Res OCT nomenclature: BM, RPE layer 2, and RPE layer 4 (High-Res OCT nomenclature).^11^ Goerdt et al^11^ reported instances in which differentiation among RPE layers 2 to 4 was not possible, suggesting that in these cases, an algorithm might depict BM as the outer RPE layer and RPE layers 2 to 4 as the middle RPE layer.^11^ Consequently, if the algorithm had misidentified BM as the outer hyperreflective RPE layer, the thickness would have been restricted to the histological thickness of BM alone (roughly 2–4 μm). Because the 95% confidence intervals for this layer thickness remained below 5 μm in every ETDRS subfield and across all age groups, such a systematic mislabeling can be considered highly unlikely. Furthermore, manual spot checks (each participant and B-scan number checked once) using the A-scan plot method revealed no such cases of outer RPE solely being BM, reinforcing the robustness and precision of the segmentation approach used.

In conclusion, this study successfully demonstrates the capability of High-Res OCT imaging to delineate multiple layers within the RPE, revealing significant age-related changes in hyperreflective bands. By establishing normative data on RPE layer thickness and reflectivity, it lays the groundwork for developing structural biomarkers for early retinal disease detection. The high segmentation accuracy achieved underscores the potential of automated RPE analysis for future clinical applications. Future work should validate these findings in retinal diseases and explore longitudinal changes in RPE integrity.
