# Supplementary material for: Automated Segmentation of Retinal Pigment Epithelium Layers in High-Resolution OCT
Source: Ophthalmol Sci. 2026 Aug 4;6(11):101354. doi: 10.1016/j.xops.2026.101354 (PMC13661213; doi:10.1016/j.xops.2026.101354)

Supplement Figure 1: Annotation of ground truth in Heyex 2

Outer retinal layers in one central B-Scan (algorithmic depiction) of a High-Res OCT image, divided in five parts to showcase segmentation steps. (A) Showcasing the 5 RPE layers as proposed by Goerdt et al[11]. (B) Heyex default segmentation line ‘BM’ (brown) was placed between the choroid and the outer hyperreflective layer. (C) Heyex default segmentation line ‘RPE’ (blue) was placed between middle and outer hyperreflective layer. (D) Heyex default segmentation line ‘PR2’ (colored orange for illustrative purposes) was first used to divide inner and middle hyperreflective layer. (E) After the download of the preliminary segmentation (described in C), segmentation line ‘PR2’ (colored green for illustrative purposes) was moved and repurposed as the top border of the inner hyperreflective layer. Layer annotations were always marked at the top of a respective layer.

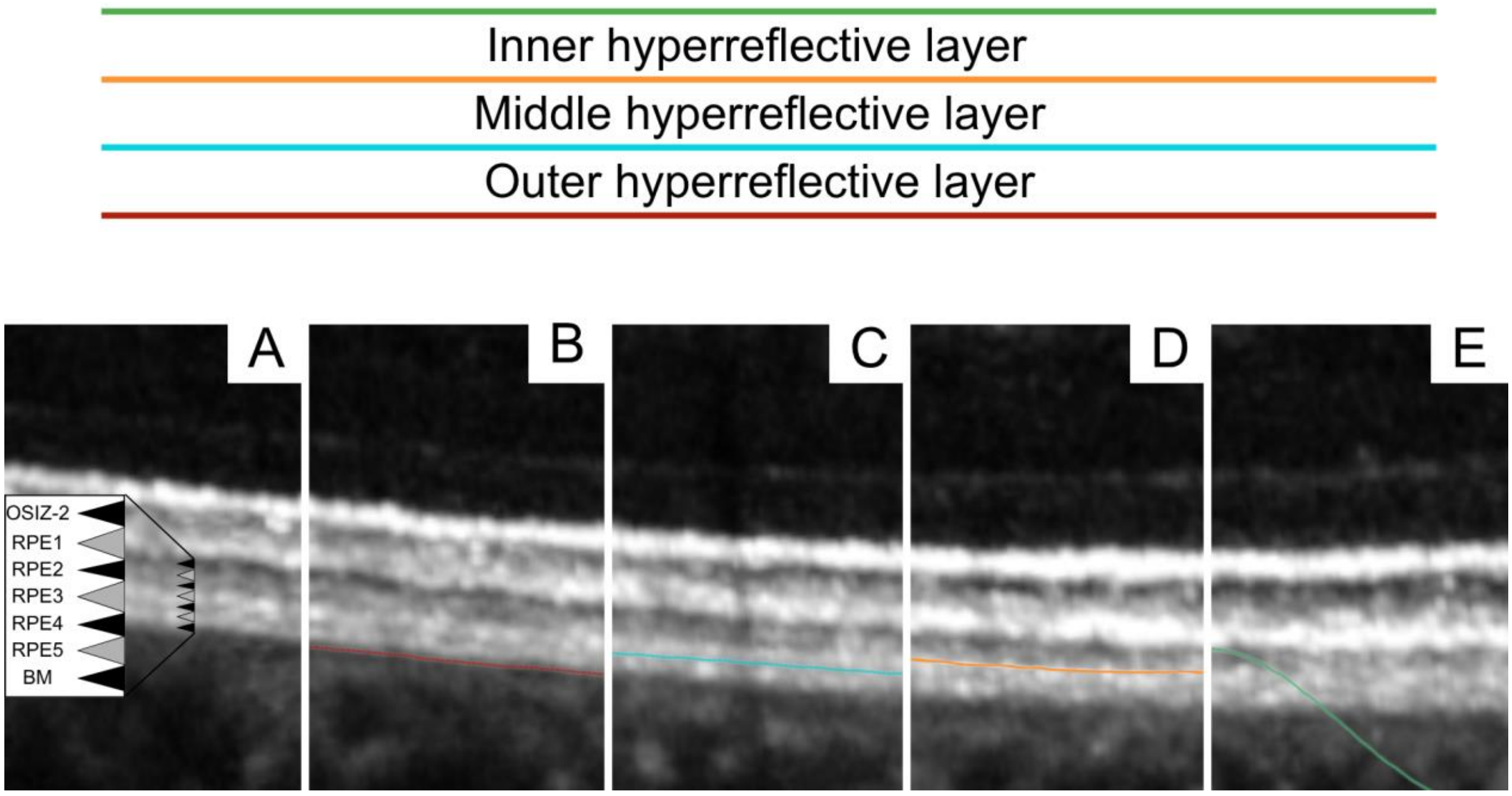

Supplement: Supplementary file 1 [file mmc1.pdf]
